# Sociodemographic characteristics associated with the utilization of maternal health services in Cambodia

**DOI:** 10.1186/s12913-020-05652-1

**Published:** 2020-08-24

**Authors:** Donghua Zhou, Zhonghe Zhou, Cheng Yang, Lu Ji, Bishwajit Ghose, Shangfeng Tang

**Affiliations:** 1 School of Physical Education, Research Center of Sports and Health, Wuhan Business University, Wuhan, 430056 Hubei P. R. China; 2 Department of Neurology, General Hospital of Northern Theater Command, Shenyang, 110840 Liaoning China; 3 School of Physical Education, Wuhan Business University, Wuhan, 430056 Hubei P. R. China; 4 School of Medicine and Health Management, Tongji Medical College, Huazhong University of Science and Technology, Wuhan, 430030 Hubei P. R. China; 5 Faculty of Social Sciences, University of Ottawa, Ottawa, Canada

**Keywords:** Cambodia, Demographic and health survey, Maternal healthcare services, Sociodemographic inequality

## Abstract

**Background:**

Cambodia is a Southeast Asian country and has one the highest rates of maternal and child mortality with inadequate use of maternal healthcare services in the region. The present study aimed to analyse the progress made in terms of using maternal healthcare services since 2000.

**Methods:**

Two rounds of Demographic and Health Surveys (DHS 2000 and DHS 2014) were used in the study. Sample population consisted 11,961 women aged between 15 and 49 years. The outcome measures were: Timing of first antenatal care (ANC) attendance, adequacy of ANC attendance, place of delivery and postnatal checkup. WHO guidelines were used to set the cut-off/define these measures. Data were analyzed in Stata version 14 using descriptive and multivariate regression analyses.

**Results:**

Findings indicated that the overall prevalence of making the first ANC visit in the first trimester was 64.19% [95%CI = 62.22,66.11], and that of having at least four ANC visits was 43.80% [95%CI = 41.89,45.73]. Prevalence of health facility delivery was 48.76% [46.62,50.90] and that of postnatal checkup was 71.14% [95%CI = 69.21,73.01]. Between 2000 and 2014, the percentage of timely and adequate use of ANC increased by respectively 61.8 and 65.3%, while that of health facility delivery and postnatal care increased by respectively 74.5 and 43.9%. Important demographic, socioeconomic and geographic disparities were observed in the utilization of ANC, health facility delivery and postnatal care services. Urban residency, having better educational status, white collar job, access to electronic media showed positive association, whereas higher parity (having > 2 children) and unwanted pregnancy showed negative association with the use of maternal healthcare services. Having at least four ANC visits was associated with significantly increased higher odds of using health facility delivery and postnatal care.

**Conclusion:**

There has a been a remarkable increase in the prevalence of women who are using the maternal healthcare services since 2000. The current findings provide important insights regarding the sociodemographic factors associated with the utilization of maternal health services in Cambodia that could contribute to evidence-based health policy making and designing intervention programs.

## Background

Cambodia is low-lying country in the Southeast Asian region, and has a population of over 16 million (as of 2019). Since obtaining independence in 1953, the country has made significant improvements in terms of reducing the percentage of absolute poverty, malnutrition, and improving the key public health indicators such as maternal and child mortality rates. Between 2000 and 2014, Cambodia managed to cut the rate of neonatal mortality by 46% [1] in parallel with an equally impressive gain in reducing maternal mortality (488 in 2000 to 160 in 2017) [2]. Despite these major advancements, the country still ranks among the least developed countries and faces critical challenges for meeting the millennium and sustainable development goals.

In recent years, healthcare system in Cambodia has implemented nationwide initiatives to promote reproductive, maternal, newborn and child health. A continuum of essential medical services such as antenatal care, skilled childbirth, and postnatal care services has been put in place in order to provide lifesaving medical services for pregnant women and their newborn. ANC services include a wide range of interventions designed to minimize obstetric complications and ensure a healthy pregnancy outcome. These services generally include anthropometric tests, blood and urine analysis, nutritional and psychological counselling, vaccination, dietary supplementation, informing the mother regarding the danger signs, mental and financial preparedness for childbirth, and importance of using skilled birthing services [3–7]. A study involving 69 low-middle countries found that even a single ANC visit could reduce the probability of neonatal and infant mortality respectively 1.04% points and 1.07% points [3]. In addition to routine ANC, WHO also emphasizes on choosing professional childbirth services and postnatal checkup (PNC) which are crucial for providing emergency obstetric services and avoiding the risks of prolonged labour, stillbirth, obstetric and postpartum hemorrhage [8–10]. These services are highly cost-effective and their proper utilization is regarded as a central strategy to reducing maternal and child mortality in all low-middle-income countries [11].

WHO recommends attending at least four antenatal visits (which was later revised to eight) during the course of the pregnancy with the first visit taking place no later than the 12 weeks from conception. Nonetheless, making these services universally available and ensuring their optimum use in an equitable manner is an extremely challenging task, especially for resource-poor countries where medical infrastructure and skilled professionals are often shockingly scarce. Geographic remoteness, high out-of-pocket costs of care, absence of skilled care provider and medium for health communication pose significant challenges for improving the use of ANC services particularly among the disadvantaged communities [12, 13]. Maternal healthcare services (MHS) such as ANC are provided through primary care clinics and community health centers in the urban and rural areas. Cambodia has been experiencing rising socioeconomic standards among women driven by higher school completion rate and engagement in labour force. Agriculture is the major economic activity in the rural areas, while in the urban areas women are participating in service and manufacturing industries. Improved socioeconomic situation constitute a key determinant of healthcare utilisation among women. Nonetheless, a large segment of the population still faces significant barriers in accessing basic health services. Addressing the barriers is crucial for promoting public health and meeting the national and international development goals targeted to reducing maternal and child mortality e.g. Millennium and Sustainable Development Goals.

ANC service in Cambodia are mainly provided through the primacy care facilities, however, midwives and community health workers also play vital roles especially in the remote areas with limited access to service [13, 14]. Large demographic and regional gaps remain in the provision and utilisation of these services as the healthcare system suffers from infrastructural and manpower crises. Understanding the population sub-groups who are being deprived from these services is an important step to scaling up the existing maternal health programs and making new evidence-based policies. In this regard, the present study attempts to provide an assessment of the progress made in terms of maternal healthcare services since 2000. We used two rounds of Demographic and Health Surveys (DHS 2000 and DHS 2014) that collected data on various demographic, socioeconomic and healthcare related indicators among women of childbearing age (15–49 years). DHS data for Cambodia were studied previously to identify the inequalities in the use of ANC and childbirth services. However, currently there is no study covering all the components of pre- (timing and frequency of ANC) and postpartum services (childbirth and postnatal care).

## Methods

### Data source

Data for this study were secondary and obtained from the Cambodia Demographic and Health Survey 2014 and 2000. The implementing organization was National Institute of Statistics (NIS – Ministry of Planning) in collaboration with the Ministry of Health. Fieldwork for the latest survey lasted from June 2014 to December 2014, and the previous one from July 2010 to January 2011. The survey received technical assistance by ICF provided through The DHS Program, a project funded by the United States Agency for International Development. This is a nationally-representative project that surveys adult men, women (15–49 years) and children under 5 years of age. The survey is cross-sectional in nature and collects data using a standard questionnaire which comprises questions covering demographic, socioeconomic, knowledge of reproductive health, and healthcare use related topics. Sampling techniques included multistage cluster approach that involved systematic selection of the geographic divisions of the country, and then the selection of households from those divisions. Detailed description of these sampling methods was published elsewhere: National Institute of Statistics, Directorate General for Health, and ICF International, 2015 [15].

### Description of study variables

The outcome variables included: 1) Timing of first ANC attendance (Within first 3 months = timely, and after 3 months = late), and 2) Total number of ANC attendance (< 4 visits = inadequate and 4 or more visits = adequate), 3) place of delivery (Home Vs health facility), and 4) postnatal checkup (Yes Vs No). The independent variables included the basic demographic and socioeconomic characteristics that were considered important for healthcare seeking behaviour. They are as follows: Age groups (15–19, 20–24, 25–29, 30–34, 35–39, 40–44, 45–49 years); Residence (Urban, Rural); Education (No Education, Primary, Secondary/higher); Education of husband (No Education, Primary, Secondary/higher); Wealth quintile (Poorest, Poorer, Middle, Richer, Richest) [16]; Household head’s sex (Male, Female); Occupation type (None, White collar, Blue collar) [17]; Decision maker about healthcare (Respondent Alone, Respondent & Partner, Others); Electronic media e.g. TV/Radio access (No, Yes); Last child wanted (No, Yes); Parity (1–2, > 2) [17–24].

### Data analysis

Data analysis consisted of descriptive statistics, chi-squared bivariate tests and multivariate analyses using Stata version 14. Firstly, datasets for 2000 and 2014 were merged and then descriptive statistics were used to calculate the prevalence of maternal healthcare utilisation for both surveys. This was followed by binary regression analysis (logit link) to measure the adjusted association between the outcome and independent variables. For multivariate analysis, only the variables that showed significance at *p* < 0.1 in the bivariate analysis were selected for multivariate analysis. For each of the four outcome variables, three sets of regression models were run: overall sample, urban and rural. The urban-rural stratification was done given the growing recognition of the regional difference in healthcare seeking and importance in designing intervention measures. Results of regression models were presented as odds ratios and 95% confidence intervals. Statistical significance was assumed at *p* < 0.05. We used Variance Inflation Factor in Stata to diagnose multicollinearity following the multivariate analyses. We did not find any such multicollinearity to report as the Variance Inflation Factor values for all the models were far below the cut-off of 10.

## Results

Table 1 summarises the prevalence of utilization of the four types of services. Sample population consisted of 11,961 women aged 15–49 years. Descriptive results indicated that a greater proportion of the women who used these services were generally aged between 25 to 34 years, rural residents, had primary level education, from higher wealth index households, male-headed households, employed in blue collar jobs, made healthcare decisions by themselves, had access to electronic media, reported last child as wanted, and had 1–2 children.

**Table 1:** Prevalence of using maternal healthcare services by sociodemographic factors (*n* = 11,961)

	Timing of ANC		Frequency of ANC		Place of delivery		Postnatal care	
	After first trimester	First trimester	Four or more	<Four	Healthcare facility	Home	Yes	No
**Age**								
15–19	4.91 [3.99,6.03]	3.10 [2.56,3.75]	3.85 [3.34,4.43]	3.17 [2.61,3.84]	3.38 [2.92,3.92]	3.71 [3.10,4.44]	3.86 [3.19,4.66]	3.28 [2.79,3.84]
20–24	18.33 [16.66,20.13]	23.94 [22.30,25.66]	16.73 [15.69,17.82]	23.28 [21.58,25.08]	15.13 [14.08,16.25]	24.32 [22.68,26.04]	15.60 [14.05,17.28]	21.45 [20.18,22.78]
25–29	23.87 [22.12,25.73]	31.43 [29.84,33.07]	24.50 [23.21,25.82]	31.36 [29.74,33.03]	24.58 [23.23,25.98]	30.61 [29.04,32.24]	25.09 [23.27,27.00]	28.46 [27.22,29.73]
30–34	25.07 [23.34,26.88]	26.24 [24.62,27.93]	23.69 [22.51,24.90]	26.75 [25.12,28.45]	23.84 [22.59,25.14]	26.25 [24.71,27.85]	23.20 [21.54,24.95]	25.53 [24.25,26.85]
35–39	16.55 [15.03,18.19]	10.22 [9.20,11.35]	17.84 [16.79,18.95]	10.12 [9.13,11.20]	18.82 [17.73,19.96]	9.87 [8.83,11.01]	18.61 [17.19,20.12]	12.86 [11.92,13.85]
40–44	9.22 [8.02,10.58]	3.93 [3.27,4.71]	10.50 [9.61,11.47]	4.32 [3.65,5.11]	11.39 [10.44,12.42]	4.00 [3.41,4.69]	10.96 [9.73,12.33]	6.56 [5.83,7.38]
45–49	2.04 [1.55,2.70]	1.14 [0.83,1.55]	2.90 [2.46,3.41]	1.00 [0.73,1.37]	2.85 [2.42,3.34]	1.24 [0.90,1.70]	2.68 [2.12,3.38]	1.87 [1.52,2.30]
**Residence**								
Urban	15.60 [12.80,18.88]	17.54 [14.58,20.96]	10.81 [8.79,13.23]	18.53 [15.48,22.02]	8.76 [6.79,11.23]	19.89 [16.70,23.51]	7.67 [5.77,10.12]	14.32 [11.99,17.01]
Rural	84.40 [81.12,87.20]	82.46 [79.04,85.42]	89.19 [86.77,91.21]	81.47 [77.98,84.52]	91.24 [88.77,93.21]	80.11 [76.49,83.30]	92.33 [89.88,94.23]	85.68 [82.99,88.01]
**Education**								
No Education	24.44 [22.14,26.90]	11.51 [10.23,12.93]	32.02 [30.09,34.00]	10.41 [9.14,11.83]	33.38 [31.25,35.57]	11.13 [9.74,12.69]	35.10 [32.65,37.64]	18.43 [16.96,20.00]
Primary	54.01 [51.68,56.33]	50.46 [48.59,52.33]	54.82 [53.08,56.54]	50.16 [48.18,52.15]	55.31 [53.37,57.23]	50.08 [48.19,51.97]	55.15 [52.74,57.53]	52.37 [50.81,53.93]
Secondary/higher	21.54 [19.56,23.67]	38.03 [35.87,40.23]	13.17 [11.95,14.49]	39.43 [37.22,41.68]	11.31 [10.07,12.69]	38.78 [36.65,40.97]	9.75 [8.44,11.24]	29.20 [27.38,31.08]
**Husband’s education**								
No Education	14.13 [12.38,16.09]	8.22 [7.17,9.43]	18.62 [17.13,20.20]	7.95 [6.89,9.14]	19.48 [17.91,21.15]	8.10 [7.01,9.35]	19.70 [17.94,21.58]	12.15 [11.03,13.37]
Primary	48.94 [46.71,51.18]	41.56 [39.61,43.55]	53.63 [51.97,55.28]	40.41 [38.41,42.44]	54.33 [52.55,56.09]	40.98 [39.00,42.99]	55.15 [53.01,57.27]	46.29 [44.58,48.01]
Secondary/higher	36.92 [34.42,39.50]	50.21 [47.97,52.45]	27.75 [26.05,29.52]	51.64 [49.30,53.98]	26.20 [24.39,28.09]	50.92 [48.57,53.27]	25.16 [23.07,27.37]	41.56 [39.56,43.58]
**Wealth quintile**								
Poorest	26.72 [24.11,29.50]	20.14 [17.69,22.85]	27.10 [24.99,29.32]	19.01 [16.66,21.61]	27.09 [24.85,29.46]	19.87 [17.51,22.46]	24.59 [22.13,27.22]	22.52 [20.38,24.82]
Poorer	24.03 [21.88,26.31]	18.97 [17.27,20.80]	21.91 [20.36,23.54]	19.09 [17.32,20.99]	21.76 [20.23,23.37]	19.55 [17.86,21.35]	24.40 [22.32,26.61]	18.98 [17.53,20.51]
Middle	18.99 [17.11,21.02]	19.50 [17.89,21.22]	20.39 [18.98,21.89]	19.44 [17.80,21.19]	20.13 [18.67,21.67]	19.79 [18.20,21.48]	20.85 [19.02,22.81]	19.52 [18.13,20.99]
Richer	15.74 [14.04,17.60]	19.33 [17.37,21.44]	16.29 [14.97,17.71]	19.91 [17.97,22.00]	16.87 [15.45,18.39]	18.99 [17.13,21.00]	16.98 [15.21,18.91]	18.48 [16.94,20.14]
Richest	14.53 [12.46,16.88]	22.06 [19.23,25.17]	14.30 [12.32,16.54]	22.55 [19.59,25.81]	14.16 [12.02,16.61]	21.81 [18.92,24.99]	13.18 [10.70,16.14]	20.50 [18.14,23.07]
**Household head’s sex**								
Male	80.54 [78.57,82.38]	78.15 [76.39,79.82]	82.39 [81.00,83.70]	78.31 [76.55,79.98]	83.78 [82.37,85.10]	77.26 [75.49,78.94]	83.71 [81.97,85.30]	79.68 [78.25,81.03]
Female	19.46 [17.62,21.43]	21.85 [20.18,23.61]	17.61 [16.30,19.00]	21.69 [20.02,23.45]	16.22 [14.90,17.63]	22.74 [21.06,24.51]	16.29 [14.70,18.03]	20.32 [18.97,21.75]
**Occupation**								
None	23.45 [21.34,25.70]	23.61 [21.28,26.13]	17.90 [16.42,19.49]	24.84 [22.45,27.38]	16.91 [15.31,18.64]	25.21 [22.89,27.67]	17.58 [15.37,20.03]	21.88 [20.06,23.83]
White collar	9.76 [8.42,11.30]	24.58 [22.13,27.20]	7.21 [6.35,8.18]	24.42 [22.07,26.94]	5.88 [4.96,6.94]	24.09 [21.79,26.55]	5.70 [4.62,7.00]	18.20 [16.43,20.12]
Blue collar	66.79 [64.28,69.21]	51.81 [49.19,54.42]	74.89 [73.03,76.66]	50.74 [48.10,53.38]	77.21 [75.17,79.13]	50.70 [48.21,53.20]	76.72 [73.98,79.26]	59.91 [57.66,62.12]
**Decision maker about healthcare**								
Respondent Alone	59.49 [56.04,62.86]	69.78 [67.19,72.26]	55.39 [52.48,58.25]	70.34 [67.67,72.88]	52.32 [49.11,55.50]	70.58 [68.10,72.95]	50.81 [46.85,54.76]	68.17 [65.74,70.51]
Respondent & Partner	36.98 [33.70,40.38]	28.04 [25.64,30.58]	40.47 [37.67,43.33]	27.52 [25.07,30.11]	43.05 [39.94,46.23]	27.36 [25.06,29.78]	44.18 [40.24,48.20]	29.57 [27.31,31.93]
Others	3.53 [2.63,4.72]	2.17 [1.60,2.93]	4.14 [3.38,5.08]	2.14 [1.55,2.93]	4.63 [3.72,5.75]	2.05 [1.54,2.73]	5.01 [3.89,6.42]	2.25 [1.75,2.91]
**Access to electronic media**								
No	20.60 [18.37,23.02]	16.43 [14.71,18.30]	24.07 [22.32,25.92]	15.83 [14.06,17.79]	24.35 [22.40,26.41]	16.35 [14.51,18.39]	27.50 [24.96,30.20]	18.67 [17.12,20.33]
Yes	79.40 [76.98,81.63]	83.57 [81.70,85.29]	75.93 [74.08,77.68]	84.17 [82.21,85.94]	75.65 [73.59,77.60]	83.65 [81.61,85.49]	72.50 [69.80,75.04]	81.33 [79.67,82.88]
**Last child wanted**								
Yes	70.18 [67.80,72.46]	84.27 [82.78,85.65]	67.46 [65.72,69.16]	84.05 [82.56,85.43]	67.23 [65.46,68.95]	82.62 [81.00,84.12]	68.62 [66.29,70.86]	77.10 [75.62,78.51]
No	29.82 [27.54,32.20]	15.73 [14.35,17.22]	32.54 [30.84,34.28]	15.95 [14.57,17.44]	32.77 [31.05,34.54]	17.38 [15.88,19.00]	31.38 [29.14,33.71]	22.90 [21.49,24.38]
**Parity**								
1–2	44.86 [42.53,47.21]	68.66 [66.81,70.46]	37.75 [36.14,39.37]	68.93 [67.08,70.72]	34.80 [33.23,36.41]	68.85 [67.16,70.48]	35.22 [33.09,37.41]	57.23 [55.57,58.87]
> 2	55.14 [52.79,57.47]	31.34 [29.54,33.19]	62.25 [60.63,63.86]	31.07 [29.28,32.92]	65.20 [63.59,66.77]	31.15 [29.52,32.84]	64.78 [62.59,66.91]	42.77 [41.13,44.43]

**Fig. 1:**
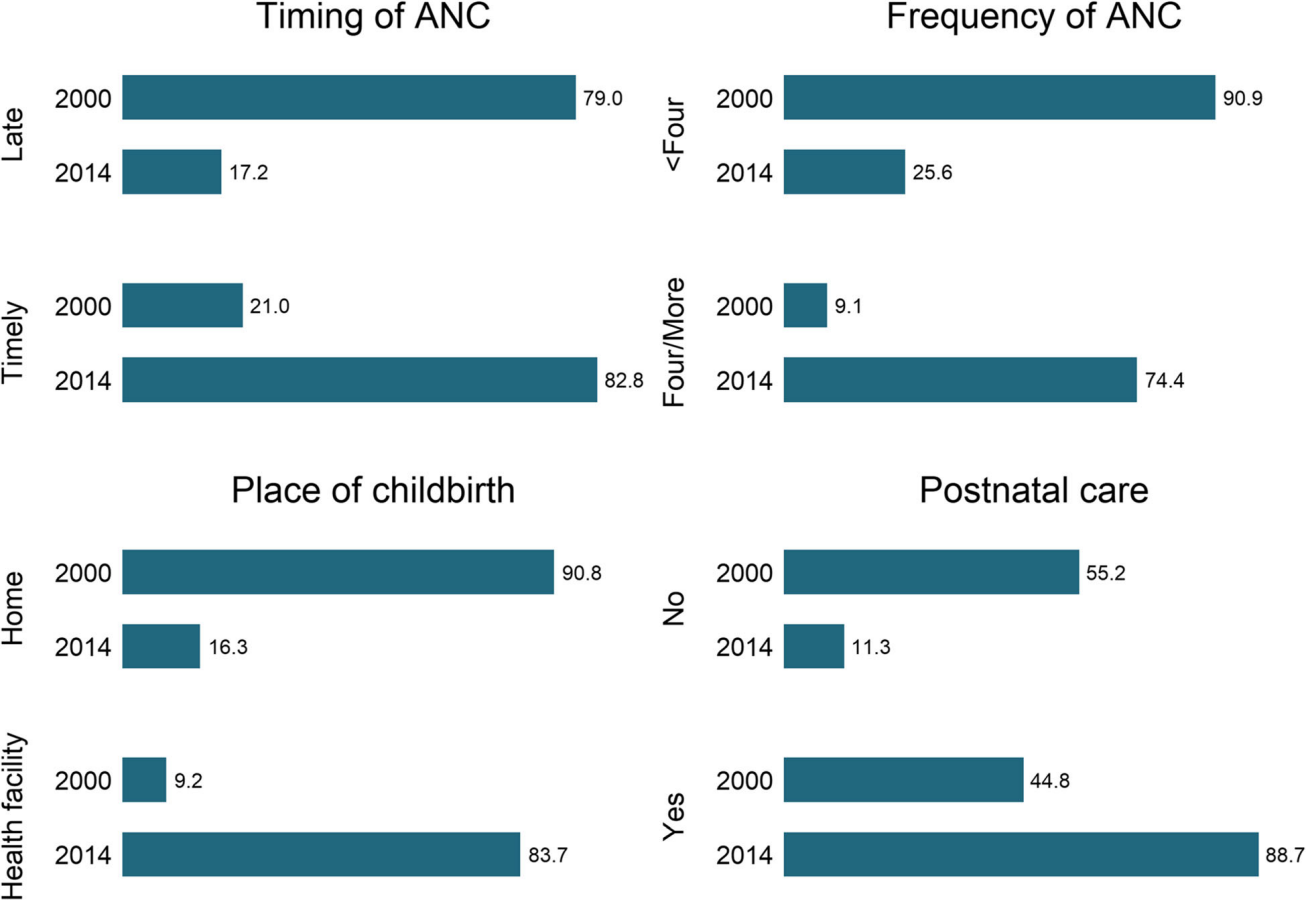
Changes in the prevalence of using maternal healthcare services between 2000 and 2014 in Cambodia (%)

Figure 1 shows that between 2000 and 2014, the percentage of timely and adequate use of ANC increased by respectively 61.8 and 65.3%, while that of health facility delivery and postnatal care increased by respectively 74.5 and 43.9%.

Sociodemographic inequalities in timely and adequate use of ANC services were presented in Table 2. Compared with 2000, the odds of timely [Odds ratio = 15.09, 95%CI = 12.39,18.38] and adequate use of ANC services [Odds ratio = 17.36, 95%CI = 14.58,20.67] increased substantially in 2014. The odds of these outcomes increased among women in the higher age groups, except for those aged > 44 years. Rural residence showed a significantly negative association with adequate use of ANC services [Odds ratio = 0.67, 95%CI = 0.55,0.82]. Increased odds ratios were also observed among women who had secondary/higher education, and the same was true when the husband had secondary/higher education as well. Wealth status didn’t show any significant association with early initiation of ANC, however, women in the middle [Odds ratio = 1.30, 95%CI = 1.05,1.62] and richer [Odds ratio = 1.49, 95%CI = 1.18,1.88] wealth quintiles were more likely to have adequate ANC visits. Women who were employed in white collar profession had significantly higher odds of timely and adequate use of ANC. Having access to electronic media was associated higher odds of making adequate ANC visits [Odds ratio = 1.45, 95%CI = 1.22,1.73]. Having unwanted child and higher parity (> 2 children) showed a negative association with both timely and adequate use of ANC services. More details were shown in Table 2.

**Table 2:** Sociodemographic characteristics associated with the use of timely and adequate use of ANC services

	ANC at first trimester			Adequate number of ANC		
	Total	Urban	Rural	Total	Urban	Rural
**Year (2000)**	ref	ref	ref	ref	ref	ref
2014	15.09^***^ (12.39,18.38)	21.35^***^ (13.23,34.45)	15.23^***^ (12.06,19.22)	17.36^***^ (14.58,20.67)	12.08^***^ (8.00,18.26)	21.47^***^ (17.41,26.49)
**Age (15–19)**	ref	ref	ref	ref	ref	ref
20–24	2.06^**^ (1.32,3.21)	1.05 (0.37,2.96)	2.41^***^ (1.46,3.95)	1.69^*^ (1.12,2.54)	3.30^**^ (1.37,7.97)	1.45 (0.91,2.30)
25–29	2.51^***^ (1.62,3.90)	1.32 (0.48,3.58)	2.89^***^ (1.76,4.73)	2.24^***^ (1.49,3.35)	3.68^**^ (1.58,8.57)	1.95^**^ (1.23,3.09)
30–34	2.29^***^ (1.46,3.59)	1.73 (0.62,4.84)	2.38^***^ (1.43,3.95)	2.71^***^ (1.79,4.12)	4.76^***^ (2.00,11.35)	2.34^***^ (1.46,3.77)
35–39	2.22^**^ (1.36,3.62)	1.47 (0.48,4.53)	2.39^**^ (1.38,4.15)	2.35^***^ (1.50,3.68)	4.47^**^ (1.72,11.59)	2.02^**^ (1.21,3.36)
40–44	1.99^*^ (1.16,3.42)	1.22 (0.35,4.26)	2.18^*^ (1.19,4.00)	2.07^**^ (1.27,3.40)	3.44^*^ (1.21,9.82)	1.86^*^ (1.06,3.26)
45–49	1.49 (0.72,3.09)	1.33 (0.19,9.22)	1.48 (0.66,3.30)	1.22 (0.64,2.33)	2.55 (0.58,11.14)	1.01 (0.49,2.09)
**Residence (Urban)**	ref	ref	ref	ref	ref	ref
Rural	0.87 (0.70,1.07)			0.67^***^ (0.55,0.82)		
**Education (None)**	ref	ref	ref	ref	ref	ref
Primary	1.26 (0.99,1.60)	2.87^***^ (1.54,5.33)	1.10 (0.85,1.42)	1.55^***^ (1.27,1.90)	1.10 (0.64,1.91)	1.66^***^ (1.34,2.07)
Secondary/higher	1.68^***^ (1.26,2.23)	3.58^***^ (1.87,6.84)	1.42^*^ (1.03,1.96)	2.39^***^ (1.87,3.06)	2.22^**^ (1.24,3.99)	2.22^***^ (1.68,2.94)
**Husband’s Education (None)**	ref	ref	ref	ref	ref	ref
Primary	1.18 (0.90,1.54)	0.97 (0.45,2.11)	1.24 (0.93,1.65)	1.35^**^ (1.08,1.69)	2.03^*^ (1.07,3.83)	1.26 (0.99,1.61)
Secondary/higher	1.51^**^ (1.13,2.03)	1.83 (0.84,4.01)	1.43^*^ (1.04,1.97)	1.96^***^ (1.53,2.52)	3.53^***^ (1.85,6.75)	1.73^***^ (1.31,2.27)
**Wealth quintile (Poorest)**	ref	ref	ref	ref	ref	ref
Poorer	0.85 (0.68,1.07)	1.08 (0.49,2.35)	0.85 (0.67,1.08)	1.10 (0.91,1.34)	0.67 (0.34,1.31)	1.15 (0.94,1.42)
Middle	1.02 (0.80,1.31)	0.94 (0.45,1.98)	1.04 (0.79,1.36)	1.30^*^ (1.05,1.62)	0.81 (0.43,1.55)	1.40^**^ (1.11,1.78)
Richer	1.01 (0.78,1.32)	0.65 (0.32,1.33)	1.13 (0.84,1.52)	1.49^***^ (1.18,1.88)	0.72 (0.39,1.34)	1.83^***^ (1.41,2.39)
Richest	1.08 (0.82,1.41)	0.73 (0.37,1.44)	1.19 (0.85,1.67)	1.19 (0.94,1.52)	0.61 (0.34,1.10)	1.60^**^ (1.19,2.16)
**Households head (Male)**	ref	ref	ref	ref	ref	ref
Female	1.03 (0.85,1.26)	1.10 (0.74,1.64)	1.03 (0.82,1.30)	0.95 (0.80,1.14)	0.90 (0.64,1.28)	0.99 (0.80,1.22)
**Employment (None)**	ref	ref	ref	ref	ref	ref
White collar	1.41^***^ (1.16,1.71)	1.37 (0.55,3.44)	1.49^**^ (1.17,1.89)	3.40^**^ (1.39,8.32)	1.60 (0.68,3.75)	11.45 (0.99,133.06)
Blue collar	1.09 (0.43,2.81)	0.80 (0.56,1.14)	1.31 (0.56,3.06)	1.13 (0.38,3.33)	0.87 (0.63,1.20)	7.81 (0.68,90.36)
**Financial decision maker (Respondent Alone)**	ref	ref	ref	ref	ref	ref
Respondent & Partner	1.10 (0.93,1.31)	0.90 (0.63,1.29)	1.15 (0.94,1.40)	0.97 (0.84,1.13)	0.92 (0.66,1.27)	1.01 (0.85,1.20)
Other	1.52 (0.44,5.30)	2.75 (0.12,61.95)	1.41 (0.35,5.68)	0.63 (0.18,2.18)	4.00 (0.31,51.95)	0.28 (0.04,2.11)
Husband/Partner Alone						
**Electronic media user (No)**	ref	ref	ref	ref	ref	ref
Yes	1.22 (0.99,1.51)	1.11 (0.55,2.25)	1.25^*^ (1.01,1.56)	1.45^***^ (1.22,1.73)	2.52^**^ (1.42,4.50)	1.38^***^ (1.14,1.66)
**Last child wanted (Yes)**	ref	ref	ref	ref	ref	ref
No	0.79^*^ (0.64,0.97)	0.62^*^ (0.40,0.95)	0.62^**^ (0.46,0.84)	0.78^**^ (0.65,0.94)	0.99 (0.67,1.47)	0.74^**^ (0.60,0.91)
**Parity (1/2)**	ref	ref	ref	ref	ref	ref
**> 2**	0.65^***^ (0.53,0.79)	0.64^*^ (0.41,0.99)	0.65^***^ (0.51,0.82)	0.51^***^ (0.42,0.61)	0.46^***^ (0.31,0.67)	0.53^***^ (0.43,0.65)
Pseudo *R*^2^	0.255	0.286	0.245	0.351	0.285	0.349

Exponentiated coefficients; 95% confidence intervals in brackets

^*^ *p* < 0.05, ^**^ *p* < 0.01, ^***^ *p* < 0.001

Sociodemographic inequalities in the use of professional childbirth and PNC services were presented in Table 3. Similar to using ANC services, the odds of professional childbirth [Odds ratio = 17.89, 95%CI = 14.55,21.99] and PNC services [Odds ratio = 6.31, 95%CI = 5.20,7.66] increased substantially in 2014 compared with 2000. Rural residence also showed a significantly negative association with professional childbirth [Odds ratio = 0.29, 95%CI = 0.23,0.38] and PNC services [Odds ratio = 0.71, 95%CI = 0.56,0.92]. Women with secondary/higher education had significantly higher odds of using professional childbirth [Odds ratio = 2.22, 95%CI = 1.67,2.95] and PNC services [Odds ratio = 1.87, 95%CI = 1.42,2.47]. These odds were higher when the husband had higher education [Odds ratio = 2.13, 95%CI = 1.60,2.83] as well. Higher household wealth quintile showed a positive association with using professional childbirth services. Having access to electronic media showed positive association with using PNC services [Odds ratio = 1.50, 95%CI = 1.25,1.80]. Having more than 2 children showed a negative association with the use of professional childbirth [Odds ratio = 0.53, 95%CI = 0.43,0.66] and PNC services [Odds ratio = 0.74, 95%CI = 0.60,0.90]. Those who had four or more ANC visits had higher odds of using professional childbirth [Odds ratio = 3.44, 95%CI = 2.90,4.09] and PNC services [Odds ratio = 2.18, 95%CI = 1.81,2.64]. More details were shown in Table 3.

**Table 3:** Sociodemographic characteristics associated with the use of professional childbirth and postnatal care services

	Facility delivery			Postnatal care		
	Total	Urban	Rural	Total	Urban	Rural
**Year (2000)**	ref	ref	ref	ref	ref	ref
2014	17.89^***^ (14.55,21.99)	10.24^***^ (6.21,16.91)	20.31^***^ (15.98,25.81)	6.31^***^ (5.20,7.66)	7.07^***^ (3.90,12.83)	6.10^***^ (4.94,7.52)
**Age (15–19)**	ref	ref	ref	ref	ref	ref
20–24	0.92 (0.56,1.51)	0.98 (0.31,3.13)	0.93 (0.54,1.60)	1.10 (0.69,1.76)	0.72 (0.20,2.55)	1.18 (0.71,1.96)
25–29	1.10 (0.67,1.79)	1.75 (0.56,5.49)	1.03 (0.60,1.76)	1.42 (0.89,2.27)	2.28 (0.63,8.23)	1.36 (0.82,2.26)
30–34	1.30 (0.78,2.15)	2.06 (0.64,6.67)	1.26 (0.72,2.20)	1.62 (,2.62)	1.46 (0.40,5.30)	1.64 (0.97,2.77)
35–39	1.13 (0.66,1.92)	2.21 (0.61,7.97)	1.06 (0.58,1.91)	1.34 (0.81,2.22)	1.13 (0.28,4.55)	1.35 (0.78,2.33)
40–44	1.23 (0.69,2.19)	2.68 (0.66,10.92)	1.16 (0.61,2.19)	1.35 (0.79,2.29)	1.01 (0.23,4.42)	1.39 (0.78,2.47)
45–49	0.78 (0.38,1.62)	1.66 (0.21,13.34)	0.73 (0.33,1.60)	1.55 (0.79,3.01)	2.30 (0.15,35.14)	1.52 (0.75,3.08)
**Residence (Urban)**	ref	ref	ref	ref	ref	ref
Rural	0.29^***^ (0.23,0.38)			0.71^**^ (0.56,0.92)		
**Education (None)**	ref	ref	ref	ref	ref	ref
Primary	1.40^**^ (1.12,1.74)	2.34^**^ (1.25,4.39)	1.29^*^ (1.02,1.64)	1.09 (0.90,1.32)	0.89 (0.46,1.74)	1.11 (0.90,1.35)
Secondary	2.22^***^ (1.67,2.95)	2.92^**^ (1.45,5.86)	2.16^***^ (1.57,2.97)	1.87^***^ (1.42,2.47)	1.51 (0.69,3.28)	1.91^***^ (1.41,2.59)
**Husband’s Education (None)**	ref	ref	ref	ref	ref	ref
Primary	1.14 (0.89,1.46)	0.73 (0.34,1.56)	1.19 (0.92,1.55)	1.21 (0.97,1.51)	1.52 (0.71,3.24)	1.19 (0.94,1.49)
Secondary	2.13^***^ (1.60,2.83)	2.16 (0.97,4.82)	2.04^***^ (1.50,2.78)	1.51^**^ (1.17,1.96)	1.50 (0.67,3.37)	1.51^**^ (1.15,1.98)
**Wealth quintile (Poorest)**	ref	ref	ref	ref	ref	ref
Poorer	1.29^*^ (1.04,1.61)	1.32 (0.63,2.75)	1.28^*^ (1.02,1.61)	0.84 (0.68,1.03)	0.58 (0.24,1.40)	0.85 (0.69,1.06)
Middle	1.40^**^ (1.09,1.78)	0.93 (0.46,1.88)	1.54^**^ (1.18,2.01)	1.01 (0.80,1.27)	1.01 (0.43,2.38)	(0.79,1.28)
Richer	1.48^**^ (1.13,1.95)	2.25^*^(1.05,4.85)	1.49^**^ (1.11,2.02)	1.17 (0.91,1.50)	1.11 (0.48,2.61)	1.17 (0.89,1.52)
Richest	1.51^**^ (1.14,2.01)	1.53 (0.81,2.90)	1.75^**^ (1.24,2.47)	1.35^*^ (1.05,1.74)	1.74 (0.83,3.67)	1.19 (0.89,1.57)
**Households head (Male)**	ref	ref	ref	ref	ref	ref
Female	1.21 (0.98,1.50)	1.36 (0.82,2.25)	1.19 (0.94,1.52)	1.04 (0.85,1.27)	1.08 (0.63,1.87)	1.03 (0.83,1.28)
**Employment (None)**						
White collar	2.23^**^ (1.25,3.97)	1.73 (0.68,4.37)	1.22 (0.96,1.55)	1.23 (0.99,1.52)	1.31 (0.92,1.87)	1.18 (0.75,1.86)
Blue collar	0.61 (0.35,1.05)	0.70 (0.43,1.13)	0.86 (0.59,1.26)	1.27 (0.81,1.99)	0.69^*^ (0.51,0.92)	0.86 (0.60,1.22)
**Financial decision maker (Respondent Alone)**	ref	ref	ref	ref	ref	ref
Respondent & Partner	0.87 (0.73,1.03)	0.81 (0.52,1.27)	0.88 (0.73,1.06)	0.87 (0.74,1.02)	0.81 (0.50,1.30)	0.89 (0.75,1.05)
Other	1.00 (0.34,2.92)	1.05 (0.08,13.73)	0.98 (0.28,3.37)	0.58 (0.25,1.36)	1.10 (0.29,4.17)	0.63 (0.27,1.48)
Husband/Partner Alone	0.51^*^ (0.29,0.91)	1.24 (0.33,4.59)	0.39^**^ (0.20,0.75)	0.51^**^ (0.32,0.83)	0.49 (0.13,1.90)	0.52^*^ (0.31,0.88)
**Electronic media user (No)**	ref	ref	ref	ref	ref	ref
Yes	1.13 (0.93,1.38)	2.82^**^ (1.49,5.33)	1.04 (0.85,1.29)	1.50^***^ (1.25,1.80)	1.72 (0.90,3.26)	1.45^***^ (1.20,1.76)
**Last child wanted (Yes)**	ref	ref	ref	ref	ref	ref
No	0.98 (0.79,1.20)	1.64 (0.96,2.83)	0.90 (0.72,1.13)	0.87 (0.73,1.05)	0.71 (0.41,1.25)	0.89 (0.74,1.08)
**Parity (1/2)**	ref	ref	ref	ref	ref	ref
> 2	0.53^***^ (0.43,0.66)	0.51^*^ (0.30,0.89)	0.53^***^ (0.42,0.67)	0.74^**^ (0.60,0.90)	1.11 (0.61,2.03)	0.70^**^ (0.56,0.87)
**ANC visits (<Four)**	ref	ref	ref	ref	ref	ref
Four/More	3.44^***^ (2.90,4.09)	3.60^***^ (2.26,5.75)	3.41^***^ (2.82,4.12)	2.18^***^ (1.81,2.64)	2.07^*^ (1.17,3.65)	2.22^***^ (1.82,2.72)
Pseudo *R*^2^	0.485	0.457	0.460	0.305	0.324	0.275

Exponentiated coefficients; 95% confidence intervals in brackets

^*^ *p* < 0.05, ^**^ *p* < 0.01, ^***^ *p* < 0.001

## Discussions

The objective of this study was to assess the situation of maternal healthcare service utilization in Cambodia. Previous studies have highlighted that services such as timing and frequency of using antenatal care, place of delivery, and postnatal checkup are vital components of MHS. Promoting the use of MHS lies at the center of attaining maternal and child health related Sustainable Development Goals. From the descriptive analysis we found that little less than two-third of the women made their first ANC visit in the first trimester, while more than two-fifth attended four or more ANC visits. Despite a relatively lower ANC attendance, we observed a relatively higher percentage of health facility delivery. The prevalence of postnatal checkup was even higher which stood at 71.14%. Although a large percentage of women are still not being able to access these life-saving healthcare services, there has been a considerable improvement in the percentage of women who used these services since 2000. Comparing the latest statistics with those from 2000 suggests that the percentage of timely and adequate use of ANC increased by respectively 61.8 and 65.3%, while that of health facility delivery and postnatal care increased by respectively 74.5 and 43.9%. The positive trend marks a major healthcare success for a low-income country like Cambodia, and provides a good example for other countries that are still lagging behind the international targets. Continued efforts will be required to further promote this success with the goal of reaching universal coverage of maternal healthcare services in Cambodia.

Further analyses were performed to identify the sociodemographic factors that are associated with the use of these services. Higher age, urban residence, better educational status, household wealth quintile (with some exceptions), employment status, access to electronic media, relatively lower parity and not having unwanted pregnancy were found to have a protective effect on the use of the healthcare services. The patterns behind these associations are in line with previous studies, and reflect the role of socioeconomic status which acts as an enabling factor for accessing healthcare, and overall health and well-being of individuals [18–20, 25, 26]. The positive role of educational status on the use of the services of the husband was particularly interesting. The absence of correlation between household wealth status with timely initiation of ANC and PNC checkup was a potentially contradictory one, although wealth status did show a significant association with having adequate ANC visits and using health facility delivery. Household financial well-being is a strong predictor of maintaining a healthy pregnancy and accessing essential medical care to ensure a successful termination of pregnancy, and health of the mother and her newborn [27–30]. This finding indicates that wealth inequality is an important factor in the use mater healthcare services. In low-income countries like Cambodia, out-of-pocket expenditure can pose serious constraints to availing life-saving medical services [5, 31, 32]. Reducing socioeconomic inequalities through patient-focused financial programs such as removal of user fees can prove beneficial to promoting the use of healthcare services and contribute to lower rates of maternal and child mortality.

Having access to electronic media showed a positive association with using adequate ANC and PNC services. Health communication through electronic media is an increasingly popular strategy to improve health knowledge and self-efficacy, which eventually contribute to more effective use of healthcare services and better population health [5, 33–36]. Regarding parity, women who had more than > 2 children were less likely to use healthcare services compared with those who had 1–2 children. The exact explanation behind this association can vary depending on individual circumstances, however, it is likely that women who experienced several pregnancies consider themselves being aware of pregnancy related health risks, and thus made healthcare visits less frequently [21, 37, 38]. Another explanation might be that the higher costs associated with having higher number of children may prevent women from seeking care. Women who reported their last pregnancy as unwanted were significantly less likely to access care. Unwanted pregnancy is a complex issue and has been shown to have negative health outcomes for both mother and children in previous studies [17, 39–41]. Healthcare strategies that can help prevent unwanted pregnancies include better provision of family planning products and counselling, especially among newly married couples. Effective use of family planning services can also increase reproductive health awareness, and thereby promote the care-seeking behaviour. Lastly, we found that women who attended four or more ANC visits were more likely to deliver at a health facility and take PNC checkup. In primary care settings, ANC services can greatly increase women’s exposure to health counselling and discussion with care providers, which enable them to make better healthcare decisions. From this view, improving ANC attendance can result in higher percentage of health facility delivery and PNC checkup, two key strategies for preventing maternal and child mortalities [42–44].

This study has several strengths and limitations to report. This study used large nationally-representative data from Cambodia Demographic and Health Survey, which is a nationally-representative survey that collects data on various domains including demographic, socioeconomic, healthcare related variables and adult men and women. The latest data were collected in 2014, and therefore may not represent the recent situation. Although the data are little old, they provide a comparative picture of the trend in maternal healthcare utlisation during the 2000–2014 period. As mentioned earlier, this was a secondary study which limited the choice of the variables or sample characteristics. For this reason, some critical variables such as cultural and infrastructure related factors were not included in the analysis. There were also no data to assess the quality of the services which are important determinants of healthcare seeking behaviour. Lastly, the surveys were cross-sectional and hence the associations are not guaranteed to establish any causal relationship. Despite these limitations, the present study provides important insights regarding the prevalence and predictors of using reproductive services which can have practical implications for reproductive health programs in the country. Further research is necessary to explore the quality of the reproductive health services in addition to merely increasing the access, to ensure sustained success in terms of maternal and child health outcomes.

## Conclusions

In this study we assessed recent progress in maternal healthcare use as well as their predictors in Cambodia. Findings indicate that little less than two-third of the women made their first ANC visit in the first trimester, more than two-fifth attended four or more ANC visits, more than half of the deliveries took place at respondent’s home, and less three quarter had PNC checkup. Year by year analysis revealed a significant improvement in the use the maternal healthcare services. Nonetheless, considerable disparities still exist in the utilisation of these services. Based on the findings, its suggestible that investing on women’s socioeconomic status, access to electronic media, and interventions targeting to reducing higher parity and unintended pregnancies may contribute to better use of maternal health services in Cambodia.

## Data Availability

Data are available at (https://www.dhsprogram.com/what-we-do/survey/survey-display-464.cfm).

## Abbreviations

DHS: Dographic and health surveys
ANC: Antenatal care
MHS: Maternal healthcare services
PNC: Postnatal checkup

## References

[1] Hong R, Ahn PY, Wieringa F, Rathavy T, Gauthier L, Hong R (2017). The unfinished health agenda: neonatal mortality in Cambodia. PLoS One.

[2] Trends in Maternal Mortality: 2000 to 2017. /featured-publication/trends-maternal-mortality-2000-2017. Accessed 3 Mar 2020.

[3] Kuhnt J, Vollmer S (2017). Antenatal care services and its implications for vital and health outcomes of children: evidence from 193 surveys in 69 low-income and middle-income countries. BMJ Open.

[4] The Public Health Importance of Antenatal Care (2015). Facts Views Vis Obgyn.

[5] Acharya D, Khanal V, Singh JK, Adhikari M, Gautam S. Impact of mass media on the utilization of antenatal care services among women of rural community in Nepal. BMC Res Notes. 2015;8. 10.1186/s13104-015-1312-8. 10.1186/s13104-015-1312-8 PMC4534014 26264412

[6] Adewuyi EO, Auta A, Khanal V, Bamidele OD, Akuoko CP, Adefemi K, et al. Prevalence and factors associated with underutilization of antenatal care services in Nigeria: A comparative study of rural and urban residences based on the 2013 Nigeria demographic and health survey. PLoS One. 2018;13. 10.1371/journal.pone.0197324. 10.1371/journal.pone.0197324 PMC5962076 29782511

[7] Anchang-Kimbi JK, Achidi EA, Apinjoh TO, Mugri RN, Chi HF, Tata RB (2014). Antenatal care visit attendance, intermittent preventive treatment during pregnancy (IPTp) and malaria parasitaemia at delivery. Malar J.

[8] Munabi-Babigumira S, Nabudere H, Asiimwe D, Fretheim A, Sandberg K (2019). Implementing the skilled birth attendance strategy in Uganda: a policy analysis. BMC Health Serv Res.

[9] Kachikis A, Moller A-B, Allen T, Say L, Chou D. Equity and intrapartum care by skilled birth attendant globally: protocol for a systematic review. BMJ Open. 2018;8. 10.1136/bmjopen-2017-019922. 10.1136/bmjopen-2017-019922 PMC5988087 29804058

[10] Onta S, Choulagai B, Shrestha B, Subedi N, Bhandari GP, Krettek A. Perceptions of users and providers on barriers to utilizing skilled birth care in mid- and far-western Nepal: a qualitative study. Glob Health Action. 2014;7. 10.3402/gha.v7.24580. 10.3402/gha.v7.24580 PMC4131000 25119066

[11] Ghose B, Feng D, Tang S, Yaya S, He Z, Udenigwe O, Ghosh S, Feng Z (2017). Women’s decision-making autonomy and utilisation of maternal healthcare services: results from the Bangladesh. BMJ open.

[12] Tang S, Ghose B, Hoque MR, Hao G, Yaya S (2019). Women Using Mobile Phones for Health Communication Are More Likely to Use Prenatal and Postnatal Services in Bangladesh: Cross-Sectional Study. JMIR Mhealth and Uhealth.

[13] In Cambodia, bringing essential care to children and mothers in remote communities. UNICEF. https://www.unicef.org/childsurvival/cambodia_92953.html. Accessed 3 Mar 2020.

[14] Ozano K, Simkhada P, Thann K, Khatri R. Improving local health through community health workers in Cambodia: challenges and solutions. Hum Resour Health. 2018;16. 10.1186/s12960-017-0262-8. 10.1186/s12960-017-0262-8 PMC5756401 29304869

[15] Zanello G, Srinivasan CS, Shankar B. What explains Cambodia’s success in reducing child Stunting-2000-2014? PLoS One. 2016;11. 10.1371/journal.pone.0162668. 10.1371/journal.pone.0162668 PMC5029902 27649080

[16] He Z, Cheng Z, Bishwajit G, Zou D (2018). Wealth inequality as a predictor of subjective health, Happiness and Life Satisfaction among Nepalese Women. Int J Environ Res Public Health.

[17] Bishwajit G, Tang S, Yaya S, Feng Z (2017). Unmet need for contraception and its association with unintended pregnancy in Bangladesh. BMC Pregnancy Childbirth..

[18] Tsala Dimbuene Z, Amo-Adjei J, Amugsi D, Mumah J, Izugbara CO, Beguy D (2018). Women's education and utilization of maternal health services in Africa: a multi-country and soioeconomic status analysis. J Biosoc Sci.

[19] Rashid M, Antai D (2014). Socioeconomic position as a determinant of maternal healthcare utilization: a population-based study in Namibia. J Res Health Sci.

[20] Zhang R, Li S, Li C, Zhao D, Guo L, Qu P, et al. Socioeconomic inequalities and determinants of maternal health services in Shaanxi Province, Western China. PLoS One. 2018;13. 10.1371/journal.pone.0202129. 10.1371/journal.pone.0202129 PMC6124721 30183720

[21] Adu J, Tenkorang E, Banchani E, Allison J, Mulay S. The effects of individual and community-level factors on maternal health outcomes in Ghana. PLoS One. 2018;13. 10.1371/journal.pone.0207942. 10.1371/journal.pone.0207942 PMC6264832 30496236

[22] Adams AM, Nababan HY, Hanifi SMMA. Building social networks for maternal and newborn health in poor urban settlements: a cross-sectional study in Bangladesh. PLoS One. 2015;10. 10.1371/journal.pone.0123817. 10.1371/journal.pone.0123817 PMC4409321 25910191

[23] Colaci D, Chaudhri S, Vasan A (2016). mHealth interventions in low-income countries to address maternal health: a systematic review. Ann Glob Health.

[24] Chi BH, Bolton-Moore C, Holmes CB (2013). Prevention of mother-to-child HIV transmission within the continuum of maternal, newborn, and child health services. Curr Opin HIV AIDS.

[25] Benova L, Campbell OM, Sholkamy H, Ploubidis GB. Socio-economic factors associated with maternal health-seeking behaviours among women from poor households in rural Egypt. Int J Equity Health. 2014;13. 10.1186/s12939-014-0111-5. 10.1186/s12939-014-0111-5 PMC4247707 25424200

[26] Novignon J, Ofori B, Tabiri KG, Pulok MH (2019). Socioeconomic inequalities in maternal health care utilization in Ghana. Int J Equity Health.

[27] Strully KW, Rehkopf DH, Xuan Z (2010). Effects of prenatal poverty on infant health: state earned income tax credits and birth weight. Am Sociol Rev.

[28] Larson CP (2007). Poverty during pregnancy: its effects on child health outcomes. Paediatr Child Health.

[29] Weck RL, Paulose T, Flaws JA (2008). Impact of environmental factors and poverty on pregnancy outcomes. Clin Obstet Gynecol.

[30] Hamad R, Rehkopf DH (2015). Poverty, pregnancy, and birth outcomes: a study of the earned income tax credit. Paediatr Perinat Epidemiol.

[31] Vora KS, Mavalankar DV, Ramani KV, Upadhyaya M, Sharma B, Iyengar S (2009). Maternal health situation in India: a case study. J Health Popul Nutr.

[32] Joshi C, Torvaldsen S, Hodgson R, Hayen A (2014). Factors associated with the use and quality of antenatal care in Nepal: a population-based study using the demographic and health survey data. BMC Pregnancy Childbirth..

[33] Asp G, Pettersson KO, Sandberg J, Kabakyenga J, Agardh A. Associations between mass media exposure and birth preparedness among women in southwestern Uganda: a community-based survey. Glob Health Action. 2014;7. 10.3402/gha.v7.22904. 10.3402/gha.v7.22904 PMC3888909 24433945

[34] Lupton D. The use and value of digital media for information about pregnancy and early motherhood: a focus group study. BMC Pregnancy Childbirth. 2016;16. 10.1186/s12884-016-0971-3. 10.1186/s12884-016-0971-3 PMC4950377 27435182

[35] O’Higgins A, Murphy OC, Egan A, Mullaney L, Sheehan S, Turner MJ (2014). The use of digital media by women using the maternity services in a developed country. Ir Med J.

[36] Zamawe COF, Banda M, Dube AN (2016). The impact of a community driven mass media campaign on the utilisation of maternal health care services in rural Malawi. BMC Pregnancy Childbirth..

[37] Sumankuuro J, Crockett J, Wang S. Maternal health care initiatives: causes of morbidities and mortalities in two rural districts of upper west region, Ghana. PLoS One. 2017;12. 10.1371/journal.pone.0183644. 10.1371/journal.pone.0183644 PMC5576685 28854248

[38] Larsen A, Exavery A, Phillips JF, Tani K, Kanté AM (2016). Predictors of health care seeking behavior during pregnancy, delivery, and the postnatal period in rural Tanzania. Matern Child Health J.

[39] Adhikari R, Soonthorndhada K, Prasartkul P (2009). Correlates of unintended pregnancy among currently pregnant married women in Nepal. BMC Int Health Hum Rights.

[40] Black AY, Guilbert E, Hassan F, Chatziheofilou I, Lowin J, Jeddi M (2015). The cost of unintended pregnancies in Canada: estimating direct cost, role of imperfect adherence, and the potential impact of increased use of long-acting reversible contraceptives. J Obstet Gynaecol Can.

[41] Trussell J (2007). The cost of unintended pregnancy in the United States. Contraception..

[42] Ameyaw EK, Kofinti RE, Appiah F. National health insurance subscription and maternal healthcare utilisation across mothers’ wealth status in Ghana. Health Econ Rev. 2017;7. 10.1186/s13561-017-0152-8. 10.1186/s13561-017-0152-8 PMC5405040 28444572

[43] Arthur E (2012). Wealth and antenatal care use: implications for maternal health care utilisation in Ghana. Health Econ Rev..

[44] Angore BN, Tufa EG, Bisetegen FS. Determinants of postnatal care utilization in urban community among women in Debre Birhan town, northern Shewa, Ethiopia. J Health Popul Nutr. 2018;37. 10.1186/s41043-018-0140-6. 10.1186/s41043-018-0140-6 PMC5909206 29673402

